# Bleeding Diathesis in Fawn Hooded Rats—Possible Implications for Invasive Procedures and Refinement Strategies

**DOI:** 10.3390/ani5020270

**Published:** 2015-04-28

**Authors:** Manon W. H. Schaap, Hugo van Oostrom, Saskia S. Arndt, Ludo J. Hellebrekers

**Affiliations:** 1 Division of Animal Welfare and Laboratory Animal Science, Department of Animals in Science and Society, Faculty of Veterinary Medicine, Utrecht University, Yalelaan 2, 3584 CM Utrecht, The Netherlands; E-Mails: M.schaap@uu.nl (M.W.H.S.); L.J.Hellebrekers@uu.nl (L.J.H.); 2 School of Veterinary Sciences, Bristol University, Langford, Bristol BS40 5DU, UK; E-Mail: hugo.vanoostrom@bristol.ac.uk

**Keywords:** fawn hooded rat, invasive procedures, bleeding disorder, anesthesia, surgery, refinement

## Abstract

**Simple Summary:**

The Fawn Hooded (FH) rat population is commonly used in biomedical research. It is widely acknowledged that the FH rat has a bleeding disorder, which may lead to abundant bleeding. However, the clinical consequences of this bleeding disorder have not been described in current literature. During our study, surgical procedures on FH rats led to an unanticipated loss of animals due to abundant bleeding. Adjustments made to minimize the impact of this disorder in animals undergoing invasive procedures are described. It is strongly recommended to take the bleeding diathesis into account when performing invasive procedures in FH rats and to apply the described refinements.

**Abstract:**

The Fawn hooded (FH) rat is commonly used in biomedical research. It is widely acknowledged that the FH rat has a bleeding disorder; leading to abundant bleedings. Although this bleeding disorder is investigated to model the storage pool defect; its impact on commonly performed invasive laboratory procedures has not yet been described. Our research group experienced clinically significant consequences of this bleeding disorder following invasive procedures (including intraperitoneal injections and neurocranial surgery) in the Rjlbm: FH stock. The clinical consequences of the surgical and anesthetic protocols applied; are described including the subsequent procedural refinements applied to minimize the impact of this disorder. It is strongly recommended to take the bleeding diathesis into account when performing invasive procedures in FH rats and to apply the suggested refinement of procedures.

## 1. Introduction

The Fawn hooded (FH) rat is widely used in biomedical research, e.g., as a model for various psychiatric disorders [1,2,3,4], (spontaneous) hypertension-associated renal failure [5,6], and blood factor studies (*i.e.*, atherosclerosis and coagulation [7,8]). Consequently, these FH rats are commonly subjected to invasive procedures, varying from injections to surgical interventions.

Several inbred substrains and outbred stocks of the FH rat are used, which differ genetically, physiologically and behaviorally [4,9]. It has been described that the platelets of FH rats have a defective secretion of coagulation mediators, which may lead to abundant bleeding in several inbred substrains [8,10,11]. Although this bleeding disorder of several FH populations has been investigated in the context of translational research (*i.e.*, to model the storage pool defect [8,12]), its (potential) impact on commonly performed invasive procedures has not been described in the literature. Recently, our research group was confronted with the clinical consequences of the bleeding disorder of one specific FH outbred stock, *i.e.*, the Rjlbm: FH, following invasive procedures. In accordance with the principle of the 3Rs (refinement, reduction, replacement) of Russell and Burch [13], we feel that it is important to publish this experience and to describe our refinement strategy.

## 2. Materials and Methods

In a recent study [14], we performed neurocranial surgery on adult male FH rats (Elevage Janvier, Le Genet St. Isle, France; denomination: Rjlbm: FH) for permanent implantation of epidural electrodes. All procedures were approved by the Animal Experiments Committee of the Academic Biomedical Centre, Utrecht, The Netherlands (DEC-DGK number 2011.I.08.079). The Animal Experiments Committee based its decision on “De Wet op de Dierproeven” (the Dutch “Experiments on Animals Act”, 1996) and on the “Dierproevenbesluit” (the Dutch “animal experiments decree”, 1996). Both documents are available online at http://wetten.overheid.nl. Further, all animal experiments followed the “Principles of Laboratory Animal Care” and refer to the Guidelines for the Care and Use of Mammals in Neuroscience and Behavioural Research (National Research Council 2003).

Rats were housed individually in clear 1500 U Eurostandard Type IV S cages, measuring 48 × 37.5 × 21 cm and were provided with bedding material (Aspen chips), *ad lib* access to food (CRM, Expanded, Special Diets Services Witham, United Kingdom) and water and carton houses (Rat Corner House, Bio Services B.V., Uden, The Netherlands). The environment was temperature—(21 ± 2 °C) and humidity (47% ± 3%) controlled with an inversed 12:12 h light-dark cycle. After an acclimatization period of three weeks [14], the animals underwent surgery for permanent implantation of epidural electrodes. The anesthetic protocol used was designed to combine adequate surgical analgesia with the possibility to antagonize anesthesia and thus promote fast recovery. Anesthesia was induced with an intraperitoneal (i.p.) injection of 0.25 mg/kg fentanyl (Fentanyl Janssen, Janssen-Cilag B.V., Tilburg, The Netherlands, 0.05 mg/mL fentanyl citrate) and 0.15 mg/kg dexmedetomidine (Dexdomitor^®^, Pfizer Animal Health B.V., Capelle a/d IJssel, The Netherlands, 0.5 mg/mL dexmedetomidine hydrochloride). After loss of the pedal reflex, the animal was endotracheally intubated, and anesthesia was maintained with isoflurane in 100% O_2_. The animals were provided with 8 mL of saline subcutaneously (s.c.) to support normal fluid balance and eye ointment to prevent drying of the cornea (Ophtosan eye ointment, AST farma B.V., Oudewater, The Netherlands, 10,000 IE vitamin A palmitate per gram). Subsequently, the animal was positioned in the stereotactic apparatus (model 963, Ultra Precise Small Animal Stereotaxic, David Kopf Instruments, Tujunga, CA, USA). Body temperature was monitored using a rectal probe thermometer and maintained at 37–38 °C with an adjustable electrically heated mattress. In addition to clinical assessment (*i.e.*, pedal reflexes), respiratory rate, heart rate, in- and expired CO_2_ and SpO_2_ were monitored continuously for assessment of anesthetic depth and anesthetic drug administration was adjusted appropriately. Following the skin incision, but prior to detachment of the periostium from the neurocranium, 3 mg/kg lidocaine solution (Alfacaine 2% plus adrenaline, Alfasan B.V., Woerden, The Netherlands, 20 mg/mL lidocaine hydrochloride and 0.01 mg/mL adrenaline) was applied to the periostium. Five small wired stainless steel screws (tip diameter 0.6 mm) were implanted epidurally over the vertex (4.5 mm caudal to bregma, 1 mm right from midline), primary somatosensory cortex (2.5 mm caudal to bregma, 2.5 mm right from midline), anterior cingulate cortex (1.5 mm rostral to bregma, 0.5 mm lateral from midline), and left and right frontal sinus (10 mm rostral to bregma, 1 mm lateral from midline). All electrodes were wired to a receptacle and fixed to the skull with antibiotic bone cement (Simplex^TM^ P bone cement with tobramycin, Stryker Nederland B.V., Waardenburg, The Netherlands, 0.5 g tobramycin per 20 g cement powder). The skin was closed in a single layer around the receptacle. Subsequently, anesthesia was antagonized with 0.05 mg/kg buprenorphine (i.p., Buprecare, AST Farma B.V., Oudewater, The Netherlands, 0.3 mg/mL buprenorphine) and 0.6 mg/kg atipamezole (i.p., Antisedan, Pfizer Animal Health B.V., Capelle a/d IJssel, The Netherlands, 5 mg/mL atipamezole hydrochloride). Anesthesia was antagonized in the animal’s home cage outside the surgery room in a separate room under red light conditions with extra oxygen supplied by a face-mask. After return of purposeful locomotion, the rat was returned to the animal housing room. Postoperative analgesia was provided with 0.05 mg/kg buprenorphine at 12 h intervals for 3 days after surgery and 0.2 mg/kg of meloxicam (s.c., Metacam, Boehringer Ingelheim B.V., Alkmaar, The Netherlands, 5 mg/mL) at 24 h intervals for 2 days after surgery.

## 3. Observations

This surgical and anesthetic protocol used was proven successful in earlier studies conducted in our lab, using in total over 200 adult male rats from different rat populations, including Wistar WU (Harlan Netherlands B.V. and Charles River, Germany) [15,16], Wistar Kyoto (WKY/KyoRj, Elevage Janvier, Le Genet St. Isle, France) [14] and Lister hooded (Charles River, Germany; manuscript in preparation). However, when applying this surgical and anesthetic protocol in the currently described 11 Rjlbm: FH rats, the bleeding disorder described in the literature for other FH substrains (from which the Rjlbm: FH is descending), proved to be clinically problematic in the Rjlbm stock. One animal died during the surgery due to a prolonged bleeding time after drilling holes in the neurocranium, and three animals were euthanized within two days after the surgery, because of multiple bleedings and severe weight loss.

During the surgery several remarkable observations were made. First off, several rats showed a blue discoloration of the scrotum, most likely due to a hemoscrotum, which developed in minutes after the i.p. injection to induce anesthesia. Notably, to our knowledge, hemoscrotums following an i.p. injection were never described in other rat strains or stocks. Although not quantified, compared to other rat populations used in our lab (e.g., Wistar WU, WKY/KyoRj and Lister hooded) the FH rats displayed prolonged bleeding times after incisions (skin and periosteum) and drilling of the holes in the neurocranium. Finally, qualitatively greater and prolonged post-surgical bleeding was observed in FH rats, compared to other rat populations used in our lab.

## 4. Refinement of the Surgical and Anesthetic Protocol

Based upon those observations, the surgical and anesthetic protocols for the Rjlbm: FH rats were refined. Firstly, i.p. injections for anesthesia induction were replaced by s.c. injections in the neck area. Secondly, dexmedetomidine was excluded from the protocol based upon its potentially hypertensive side effect [17]. Instead, anesthesia was induced with 3 mg/kg midazolam (Midazolam, Actavis B.V., Baarn, The Netherlands, 5 mg/mL midazolam), 0.25 mg/kg fentanyl (Fentanyl Janssen, Janssen-Cilag B.V., Tilburg, The Netherlands, 0.05 mg/mL fentanyl citrate) and 10 mg/kg ketamine (Narketan, Vétoquinol S.A., Lure Cedex, France, 115.34 mg/mL ketamine hydrochloride). Thirdly, to minimize bleeding during the surgery, after detachment of the periostium, bleeding from the neurocranium was coagulated with 30% H_2_O_2_. Finally, to support the postoperative nutritional restoration, until three days after surgery, the rats received nutritional support using Convalescence Support (Royal Canin B.V., The Netherlands), containing a high level of energy and protein concentration. Otherwise, the surgical and other aspects of anesthetic protocols were unchanged.

The refined surgical and anesthetic protocols were used in six FH rats. No further bleeding diatheses occurred. One rat immediately died after induction of anesthesia (presumed anesthesia-related problems), before the start of the surgical procedure. The remaining five rats recovered without any complications.

Further refinement of the protocol could be considered during future studies. To completely avoid injections, a protocol using volatile induction and maintenance and oral administration of analgesics (e.g., [18]) administered pre-operatively could be an option. Post-operative analgesia can also be provided by the exclusive per os route, further minimizing the need of injections.

## 5. Conclusions and Recommendation

In conclusion, the bleeding disorder of the adult male Rjlbm: FH rat, as experienced in our laboratory, may have a substantial impact when performing invasive procedures. Although invasive procedures in this specific stock can be performed, the bleeding diathesis is potentially clinically problematic and life threatening and demands special attention. This is the first report on the clinical implications of the FH’s bleeding disorder, using the Rjlbm: FH stock. To what degree other FH substrains and stocks are clinically affected by the bleeding disorder during invasive procedures in the laboratory is yet unknown. However, it is strongly recommended to take the bleeding diathesis into account when performing invasive procedures in any FH population. Applying refinement strategies, as described here, will contribute to the reduction of the number of animals and their discomfort, in accordance with the principle of the 3Rs [13] regarding laboratory animals.
